# Conversion of a Failed Vertical Banded Gastroplasty to One-Anastomosis Gastric Bypass in a Case with Gastro-gastric Fistula and Mesh Complication: A Multimedia Article

**DOI:** 10.1007/s11695-025-08069-z

**Published:** 2025-08-06

**Authors:** Mohamed Hany, Mohamed H. Zidan, Mona S. Youssef, Anwar Ashraf Abouelnasr, Nour Zayed, Mohamed Ibrahim, Aly Elbahrawy, Bart Torensma

**Affiliations:** 1 Alexandria University, Alexandria, Egypt; 2 King Abdullah Medical City, Makkah al Mukarramah, Saudi Arabia; 3 Department of Clinical Epidemiology, Erasmus MC, Rotterdam, Netherlands; 4 Department of Surgery, Medical Research Institute, Alexandria University, Alexandria, Egypt; 5 Bariatric Surgery Unit, Madina Women’s Hospital, Alexandria, Egypt; 6 The Research Papyrus Lab, Alexandria, Egypt; 7 Maidstone and Tunbridge Wells NHS Trust, Barming, Maidstone, United Kingdom

**Keywords:** Revisional metabolic bariatric surgery, Vertical banded gastroplasty, One-anastomosis gastric bypass, Gastro-gastric fistula, Mesh complication

## Abstract

A 56-year-old female presented with recurrent weight gain (RWG) and vomiting, 10 years after open VBG. Imaging and endoscopy revealed an anatomically distorted pouch, two staple line dehiscences, and a gastro-gastric fistula (GGF). Multiple revisional options were evaluated. Due to the presence of mesh and anatomical limitations, a decision was made to perform a one-anastomosis gastric bypass (OAGB) below the mesh, guided by a calibration tube passed through the area of disruption. Laparoscopic OAGB was performed with a 150-cm biliary limb and a 3-cm gastrojejunostomy. Intraoperative leak testing was negative. The patient had an uneventful recovery and was discharged on postoperative day 3. At 18-month follow-up, she achieved a weight of 74 kg (body mass index (BMI) 25.4 kg/m^2^), with complete symptom resolution and normal laboratory parameters.

**Supplementary Information:**

The online version contains supplementary material available at 10.1007/s11695-025-08069-z.

## Background

Vertical banded gastroplasty (VBG) gained popularity in the late twentieth century as a purely restrictive metabolic bariatric surgery [1–3]. Despite early success, long-term follow-up has revealed high failure rates [3, 4]. Up to 50% of patients experience substantial recurrent weight gain (RWG) and approximately 65% ultimately require revisional surgery due to complications or suboptimal weight loss [3, 4]. These late complications include stomal stenosis, staple-line dehiscence, band erosion, and gastro-gastric fistula formation [3, 4]. Many patients report persistent vomiting, reflux, and dysphagia, even in the absence of apparent anatomical failure [3–5]. Such symptoms tend to improve following revisional interventions [3, 5]. However, reversal alone is often insufficient to prevent further RWG or improve long-term associated medical problems. Thus, conversion to a more durable metabolic bariatric surgery (MBS) is widely recommended [3, 5, 6]. However, conversional options are tailored to address mesh-related complications, including fibrosis, erosion, and tissue distortion, which may complicate revisional surgery by compromising staple-line integrity [7].

Several revisional options have been explored, each with distinct limitations. Conversion to adjustable gastric banding has shown minimal additional benefit and is associated with continued risk of suboptimal weight loss, band erosion, reflux, and dysphagia [8, 9]. Sleeve gastrectomy (SG) is technically feasible but carries significant risks, particularly leakage along the fibrotic staple line, and may necessitate further revision [6].

Roux-en-Y gastric bypass (RYGB) and one anastomosis gastric bypass (OAGB) [3, 5] are among the more favored revisional procedures. RYGB remains the most frequently performed and well-studied revisional procedure following VBG, with consistent evidence supporting its safety, durability of weight loss, and effective resolution of reflux and food intolerance [7, 10, 11]. It also mirrors the outcomes of primary RYGB regarding weight loss and improvement of associated medical problems [4, 7, 11, 12]. Alternatively, OAGB has emerged as a promising revisional approach [13–15], where a low-lying mesh is present in the index surgery, permitting the creation of a long gastric pouch.

Recent data support the application of a 150-cm biliopancreatic limb not only in primary OAGB, but also in revisional procedures [16]. Petrucchi et al. evaluated 215 patients who underwent conversion from failed adjustable gastric banding to OAGB with a 150 cm BPL [16]. At 2 years, they achieved an 88% excess weight loss (EWL) and a 38.7% total weight loss (TWL), with sustained results at 5 years (82% EWL, 36% TWL). This reinforces that a fixed 150-cm BPL in revisional OAGB delivers durable weight loss, a favorable safety profile, and nutritional balance, echoing its merits in primary surgery [17, 18].

Current literature suggests that OAGB effectively reduces weight and resolves symptoms in patients with failed VBG [14, 15]. A recent meta-analysis by Kermansaravi et al. [14] further supports this, demonstrating that revisional OAGB achieves outcomes comparable to RYGB in terms of excess weight loss (66–74%) and remission of associated medical problems while potentially offering a lower complication rate (7.6%), with a low incidence of marginal ulceration and bile reflux [14].

This multimedia article presents a complex case of unsuccessful VBG, characterized by partial staple-line disruption, a gastro-gastric fistula, and an anatomically distorted pouch.

## Case Presentation

A 56-year-old female patient underwent an open VBG 10 years ago. Initially, she achieved optimal weight loss but later experienced RWG and sought advice for a revisional procedure. At the time of the original surgery, her weight was 140 kg, her height was 170 cm, and her body mass index (BMI) was 48.4 kg/m^2^. When she presented, her weight was 128 kg with a BMI of 44.2 kg/m^2^. She had previously lost a maximum of 56 kg following the VBG. Furthermore, the patient complained of infrequent vomiting.

The patient had no documented associated medical problems such as diabetes, hypertension, or obstructive sleep apnea at presentation. Her primary complaints were RWG and postprandial vomiting, with a desire to improve her overall well-being and functionality.

Her diet was unhealthy, characterized by excessive consumption of sweets. Her surgical history was otherwise unremarkable. Her laboratory workup was normal. A multidisciplinary team (MDT) consultation, including a behavioral therapist and a dietitian, was conducted to clarify the patient’s goals regarding her complaints and to establish an appropriate management plan. The patient’s main issues were RWG and vomiting, and she expressed a desire to improve her quality of life.

Investigations revealed the following:

1. Computer tomography (CT) with 3D reconstruction showed staples along the gastric cardia and lesser curvature, with a distortion noted in the mid-region. The gastric pouch appeared non-functional and compressed and was seen communicating with the distended residual stomach, suggesting a gastro-gastric fistula. The gastric pouch volume was measured at 4.9 cc, and the estimated residual gastric volume was 1134 cc.
2. Preoperative endoscopy revealed a small proximal pouch with evidence of gastritis. A narrow segment at the mesh site was impassable to the scope. Staples from the previous VBG were also noted. Two areas of distortion were identified: one just below the gastroesophageal junction, communicating with the distal stomach, and another in the mid-portion of the staple line, which prevented further advancement of the scope despite multiple attempts.

Surgical options discussed with the patient included laparoscopic LS, RYGB, OAGB, and duodenal switch (DS) as potential revisional procedures. Each option was explained in detail, including its advantages and possible complications. It was also agreed that the surgical plan might be modified intraoperatively based on the actual findings, and the patient consented.

## Surgical Technique

In June 2023, the patient was admitted for laparoscopic exploration using five ports. Adhesiolysis was performed, and a 40 Fr calibration tube was inserted through the disrupted staple line, as it could not pass through the mesh site.

Mesh removal was deemed inadvisable due to its low-lying, fibrotic positioning and proximity to the gastroesophageal junction. The procedure would have required significant dissection, elevating the risks of bleeding, tissue devascularization, or unintentional injury. In light of the absence of mesh erosion or infection as confirmed by imaging and endoscopy, coupled with the feasibility of creating a new pouch distal to the mesh, the surgical team chose to leave the mesh in situ (Video 1).

**Fig. 1:**
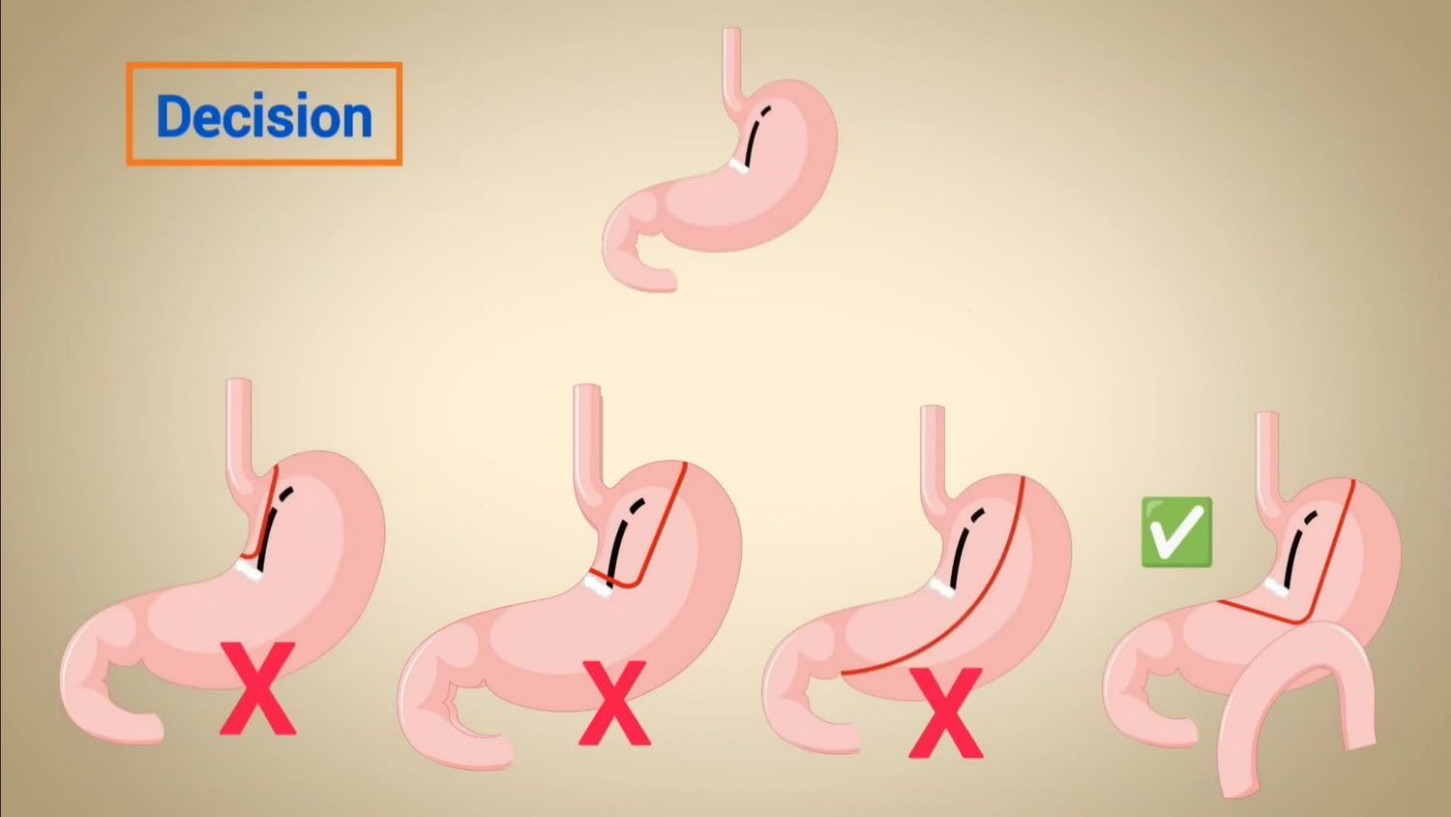
Several scenarios discussed by the surgical team

Discussion with the surgical team involved several scenarios (Fig. 1). At first glance, it appeared that the pouch could be fashioned for a RYGB. However, considering the disrupted gastric pouch, the inability to pass either the bougie or the endoscope beyond the mesh site, and the findings from radiological and endoscopic examinations, creating an RYGB pouch above the mesh was deemed unfeasible due to the distorted anatomy. This approach would have required crossing staple lines and dealing with a very narrow pouch, which could have potentially resulted in a technically unsound reconstruction.

SG and DS were excluded due to the mesh’s proximity to the intended staple line, which heightened the risk of tissue ischemia and complications related to anastomosis. The mesh is positioned near the anticipated staple line for the sleeve. Even if the reconstruction was adjusted to remain outside the compromised staple line, the intervening “island” of tissue could become ischemic or lead to mucus retention, both of which could elevate the risk of leakage.

Reconstructing a long pouch away from the disrupted staple line, guided by a bougie passed through the disruption, was also considered. However, this approach risked creating a pouch that was wider proximally and narrower distally, as the bougie would be deflected against the lesser curvature of the stomach. This configuration could result in an unsound pouch with increased tension due to pressure build-up at the incisura and food being forced to pass through the disruption over a longer distance. Such a reservoir would not effectively support weight loss, could worsen RWG, and might even exacerbate vomiting, one of the patient’s primary complaints.

The final decision was to proceed below the mesh, creating a pouch guided over a bougie passed through the area of distortion. OAGB was chosen over RYGB, prioritizing the simplicity of a single anastomosis and shorter operative time, rather than performing RYGB with an expanded long pouch.

Pouch creation was initiated by horizontally dividing the stomach below the level of the mesh. Although indocyanine green (ICG) fluorescence angiography can be a useful adjunct to assess gastric pouch perfusion, it was not employed in this case due to its unavailability. The surgical team relied on conventional visual cues, including healthy tissue color, capillary bleeding, and turgor, which collectively confirmed adequate vascularity of the gastric pouch and anastomotic site.

Furthermore, intraoperative endoscopy confirmed patency of the newly created pathway. A 3-cm gastrojejunostomy was then constructed using a linear stapler with a 150-cm biliopancreatic limb.

The gastroenterostomy was closed in two layers with hand-sewn 3-0 PDS barbed sutures (V-Loc, Medtronic, USA). The new gastric staple line was also oversewn. An additional leak test using methylene blue was performed, yielding negative results. The remaining portion of the stomach was excised to prevent the formation of a blind pouch and potential gastro-gastric fistula. A tube drain was placed. The total operative time was 125 min.

## Postoperative Course

The patient remained hemodynamically stable and was able to tolerate oral fluids. An oral contrast-enhanced CT scan on postoperative day 2 revealed an intact anastomosis with a small blind pouch and no evidence of leakage. The patient was discharged on postoperative day 3 with instructions to follow a liquid diet for 2 weeks.

At the 18-month postoperative follow-up, the patient had achieved a satisfactory weight of 74 kg with a BMI of 25.4 kg/m^2^, and laboratory findings were within normal limits. The frequency of vomiting was associated with rapid eating habits.

## Conclusion

In some instances, disruption of the staple line after VBG and abnormal configuration with recurrent weight gain can be effectively managed by conversion to a one-anastomosis gastric bypass. The management of failed VBG cases should be individualized and tailored on a case-by-case basis. Sharing clinical experiences among metabolic bariatric surgeons is encouraged to address these complex situations and to achieve the best possible outcomes for patients, even in the most challenging scenarios.

## Supplementary Information

Below is the link to the electronic supplementary material.

Supplementary file1 (mp4 150 MB)

## Data Availability

No datasets were generated or analysed during the current study.
